# Anti-Inflammatory Effect and Mechanism of the Green Fruit Extract of *Solanum integrifolium* Poir.

**DOI:** 10.1155/2014/953873

**Published:** 2014-07-15

**Authors:** Lisu Wang, Shu-Yuan Chiou, Yi-Ting Shen, Fu-Tsun Yen, Hsiou-Yu Ding, Ming-Jiuan Wu

**Affiliations:** ^1^Department of Food Science and Technology, Chia Nan University, Tainan 717, Taiwan; ^2^Crop Improvement Section, Hualien District Agricultural Research and Extension Station, Hualien 97365, Taiwan; ^3^Department of Pharmacy, Chia Nan University of Pharmacy and Science, Tainan 717, Taiwan; ^4^Department of Biotechnology, Chia Nan University of Pharmacy and Science, Tainan 717, Taiwan; ^5^Department of Cosmetic Science, Chia Nan University of Pharmacy and Science, Tainan 717, Taiwan

## Abstract

The green fruit of *Solanum integrifolium* Poir. has been used traditionally as an anti-inflammatory and analgesic remedy in Taiwanese aboriginal medicine. The goal of this study is to evaluate the anti-inflammatory activity and mechanism of the green fruit extract of *S. integrifolium*. A bioactivity-guided fractionation procedure was developed to identify the active partition fraction. The methanol fraction (ME), with the highest phenolic content, exhibited the strongest inhibitory effect against LPS-mediated nitric oxide (NO) release and cytotoxicity in RAW264.7 macrophages. ME also significantly downregulated the expression of LPS-induced proinflammatory genes, such as iNOS, COX-2, IL-1*β*, IL-6, CCL2/MCP-1, and CCL3/MIP1*α*. Moreover, ME significantly upregulated HO-1 expression and stimulated the activation of extracellular-signal-regulated kinase 1/2 (ERK1/2). Pretreatment of cells with the HO-1 inhibitor zinc protoporphyrin and MEK/ERK inhibitor U0126 attenuated ME's inhibitory activity against LPS-induced NO production. Taken together, this is the first study to demonstrate the anti-inflammatory activity of green fruit extract of *S. integrifolium* and its activity may be mediated by the upregulation of HO-1 expression and activation of ERK1/2 pathway.

## 1. Introduction

Scarlet Eggplant (*Solanum integrifolium *Poir.), native to Africa, was introduced into Taiwan long time ago. It is now recognized as an indigenous medicinal vegetable eaten by many aborigines. The fruit is harvested before it ripens and turns red and is used as an anti-inflammatory and analgesic remedy to alleviate edema or cure stomach pain, lymphadenopathy, or sore armpits in indigenous medicine. Although the green fruit of* S. integrifolium* is widely consumed, no systematic reports have yet been carried out regarding its medicinal activity.

Inflammatory responses are typically present as a series of vascular and cellular reactions initiated by injury or infection. Macrophages are in the first line of defense against invading pathogens in tissues. When stimulated by pathologic stimuli or injury, macrophages release chemokines, such as CCL2/MCP-1 and CCL3/MIP1*α*, proinflammatory cytokines, such as TNF-*α*, IL-1*β*, and IL-6 [1], and inflammatory mediators, such as prostaglandins (PGs) and nitric oxide (NO) to augment the host's defense against invasion by microbes [2–4]. Although inflammation is an intrinsically beneficial event that leads to the removal of offending factors and restoration of tissue structure and physiological function, sustained and chronic inflammation, a process associated with elevated levels of various cytokines and reactive oxygen species (ROS), has been implicated in the pathogeneses of arthritis, cancer, and stroke, as well as in neurodegenerative and cardiovascular diseases [4].

To better understand the therapeutic use of* S. integrifolium*, we analyzed the antinitric oxide activities of the crude ethanol extract and different partition fractions. The fraction with the highest polyphenolic content was further used to study the molecular mechanism underlying its anti-inflammatory activity.

## 2. Materials and Methods

### 2.1. Materials

The fresh green fruit of* S. integrifolium* was obtained from the Hualien District Agricultural Research and Extension Station in July 2013, and the voucher specimens (number SI-2013-001) were deposited in the herbarium of Chia Nan University of Pharmacy and Science. U0126 (1,4-diamino-2,3-dicyano-1,4-bis [2-aminophenylthio]butadiene), a selective and potent inhibitor of MEK activity and activation of ERK1/2, was purchased from Promega (Madison, WI). SP600125 (anthra[1,9-*cd*]pyrazole-6 (2*H*)-one), an inhibitor of c-Jun NH_2_-terminal kinase (JNK), was purchased from Calbiochem (San Diego, CA). HO-1 inhibitor zinc protoporphyrin IX (Znpp) and other chemicals were purchased from Sigma-Aldrich Co. (St. Louis, MO) unless otherwise indicated.

### 2.2. Extraction and Partition of the Green Fruit of* S. integrifolium*

The fresh green fruit were cut into small pieces, dried at 50°C, and powdered. The dry sample powder (1.2 kg) was extracted with ethanol (20 L × 3) at room temperature. The combined ethanol extract was concentrated* in vacuo* to yield dark-brown syrup (280 g). A small portion of the resin was dissolved in ethanol and denoted as the crude ethanol extract (SI).

The rest of the brown resin was resuspended in H_2_O and partitioned with an equal volume of ethyl acetate (EA) three times to yield the EA and the aqueous layers. The combined EA layer was concentrated in a rotary evaporator at 55°C, and the resulting residue was dissolved in methanol and partitioned with an equal volume of* n*-hexane three times to yield the methanol and* n*-hexane fractions.

The rest of the aqueous layer was further partitioned with an equal volume of* n*-butanol for three times to give the* n*-butanol and aqueous layers. The combined* n*-butanol layer was concentrated in a rotary evaporator at 70°C, and the resulting brown resin was dissolved in DMSO and denoted as the* n*-butanol fraction. The aqueous layer was lyophilized, and the resulting powder was dissolved in water and denoted as the water fraction.

### 2.3. Folin-Ciocalteu Assay

Total phenolic content was determined by the slightly modified Folin-Ciocalteu (F-C) assay [5]. Eight *μ*L of F-C reagent and 20 *μ*L of the proper dilution of test sample were added to each well of a 96-well plate. The contents of the wells were pipetted up and down to mix and then allowed to stand for 10 min. After this, 200 *μ*L of a 2% aqueous sodium carbonate solution was added to each well. The contents of the wells were mixed and incubated at room temperature for 10 min. Absorbance was read at 620 nm using an ELISA reader. The results are expressed as milligrams of gallic acid equivalent per gram of dry weight (mg GAE/g dw).

### 2.4. Cell Culture

RAW264.7 cells were purchased from the Bioresource Collection and Research Center (Hsinchu, Taiwan) and cultured in Dulbecco's modified Eagle's medium (DMEM) with 10% fetal bovine serum (HyClone, Logan, UT), 2 mM glutamine, 1% nonessential amino acid, 1 mM pyruvate, 100 U/mL penicillin, and 100 *μ*g/mL streptomycin (Invitrogen Life Technologies, Carlsbad, CA). Cells were maintained in a humidified incubator at 37°C in 5% CO_2_.

### 2.5. Nitrite Measurement

Nitrite production, an indicator of nitric oxide (NO) synthesis, was determined by the Griess reaction. The supernatant of cell cultures was mixed with an equal volume of Griess reagent (1% sulphanilamide and 0.1% naphthalenediamine in 5% phosphoric acid). Absorbance was read at 550 nm and calculated against a sodium nitrite standard curve.

### 2.6. Cell Viability

Cell viability was assessed by the mitochondrial-dependent reduction of 3-(4, 5-dimethylthiazol-2-yl)-2, 5-diphenyltetrazolium bromide (MTT) to purple formazan [6].

### 2.7. Western Blotting Analysis

Confluent RAW264.7 cells (1 × 10^6^/mL) in a 10 cm petri dish were incubated with either LPS (10 ng/mL;* E. coli* 055:B5, Fluka Chemie, Buchs, Switzerland) alone or LPS plus the methanol fraction (0.05–0.25 mg/mL). After being incubated for indicated period, cells were washed with PBS, scraped in ice cold RIPA buffer (Thermo Fisher Scientific Inc., Rockford, IL), and incubated on ice for 30 min. The cellular debris was removed by centrifugation (8,000 ×g for 15 min) at 4°C and the cell lysate was carefully transferred to the microcentrifuge tube. The protein concentration was measured by the Bradford method (Bio-Rad Laboratories, Hercules, CA, USA) using bovine serum albumin as a standard.

Cell lysates were separated on 8–12% SDS-PAGE and transferred onto Hybond-P PVDF (GE Healthcare) at 20 volt overnight at 4°C. The membranes were blocked at 4°C in PBST blocking buffer (5% BSA in PBS with 0.05% Tween 20, pH 7.4) for 8 h. Blots were analyzed with each primary antibody (Table 1) at a dilution of 1 : 1000–1 : 5000 overnight at 4°C. After three washes with PBST, the blots were incubated with suitable horseradish peroxidase-conjugated secondary antibody (Jackson ImmunoResearch, West Grove, PA) at a dilution of 1 : 10,000–1 : 25,000 for 1 h. The blots were washed again and the proteins of interest were detected using Amersham ECL Prime Western Blotting Detection Reagents (GE Healthcare) according to the manufacturer's instructions, and the chemiluminescence signal was then visualized with X-ray film. For reprobing, blots were treated with Restore stripping solution (Thermo Scientific).

### 2.8. RNA Extraction and Reverse Transcription Real-Time PCR

Total cellular RNA was prepared using an Illustra RNAspin Mini RNA Isolation Kit (GE Healthcare, Buckinghamshire, UK). Reverse transcription was carried out using 0.8 *μ*g RNA and High-Capacity cDNA Archive kit (Applied Biosystems, Foster City, CA, USA). Quantitative PCR was performed with 2 *μ*L of the cDNA obtained above in a 25 *μ*L solution containing 200 nM primers (Table 2) and Power SYBR Green PCR Master Mix (Life Technologies). Amplification was conducted in an ABI StepOne Real-Time PCR System. PCR conditions were as follows: 95°C for 2 min, 40 cycles at 94°C for 15 s, and 60°C for 60 s. Target gene expression was determined using the ΔΔCT method and was normalized to the respective *β*-actin expression level. The identity and purity of the amplified product was checked through analysis of the melting curve carried out at the end of the amplification process.

### 2.9. Statistical Analysis

All experiments were repeated at least three times. The results were presented as means ± SD and analyzed by Kruskal-Wallis test. A *P* value of < 0.05 was taken to be significant. If the Kruskal-Wallis test shows a significant difference between the groups, then pairwise comparisons were used by employing the Mann-Whitney *U* Tests.

## 3. Results

### 3.1. Total Phenolic Contents of Crude Extract and Different Fractions of* S. integrifolium*

**Figure 1:**
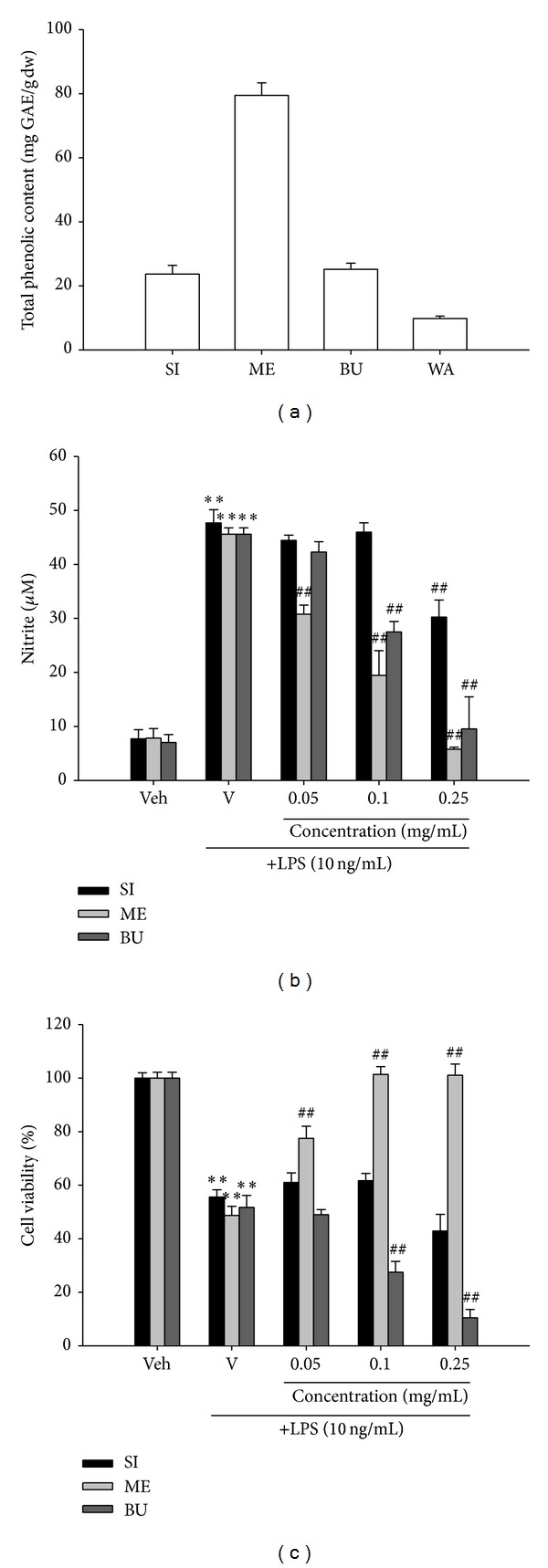
Total phenolic contents, inhibitory activities against nitric oxide release, and cytoprotective effects of* Solanum integrifolium* extract and fractions. (a) Total phenolic contents were measured using Folin-Ciocalteu reagent. (b and c) RAW264.7 macrophages were cultured with ethanol extract (SI), methanol fraction (ME), or* n*-butanol fraction (BU) at the indicated concentrations in the presence of LPS (10 ng/mL) at 37°C for 20 h in a 96-well plate. The nitrite production, an indicator of nitric oxide (NO) synthesis, was determined by the Griess reaction and the cell viability was analyzed by MTT assay. Data represent the mean ± SD of three independent experiments. **P < 0.01 represents significant differences compared with the vehicle control (without LPS). ^##^ *P* < 0.01 represents significant differences compared with the LPS-treated vehicle.

Total phenolic contents of crude ethanol extract and different fractions, namely,* n*-hexane, methanol,* n*-butanol, and water fractions, were determined using Folin-Ciocalteu reagent as described in Section 2. It is found that methanol fraction (ME) has the highest phenolic content, followed by* n*-butanol fraction (BU) and crude ethanol extract (SI), while water fraction (WA) has the lowest phenolic content (Figure 1(a)). There is no detectable phenolic content in the* n*-hexane faction.

### 3.2. Effects of Crude Ethanol Extract (SI) and Different Fractions on LPS-Induced Nitrite Oxide (NO) Production and Cytotoxicity in RAW264.7 Cells

As a model of macrophage activation, murine macrophage-like RAW264.7 cell line stimulated with lipopolysaccharide (LPS) was used. These cells have been used previously to characterize the action of various anti-inflammatory components at the molecular level [7–9]. To study whether the crude ethanol extract of* S. integrifolium* (SI) and fractions can function as inhibitors for nitric oxide (NO) release in this system, RAW264.7 cells were stimulated with LPS (10 ng/mL) and the vehicle, crude extract or fraction (Figure 1(b)). Stimulation of cells with LPS (10 ng/mL) for 20 h induced a significant increase in nitrite production from the basal level 7.7 ± 1.7 to 47.7 ± 2.4 *μ*M and 45.6 ± 1.2 *μ*M for 0.1% ethanol vehicle control and 0.1% DMSO vehicle control, respectively. ME evoked strongest inhibition against LPS-induced nitrite release, followed by BU, and SI had the weakest inhibitory activity. There was no detectable nitric oxide inhibitory activity for* n*-hexane or water fraction.

It has been reported that LPS induces apoptosis in macrophages mainly through production of cytokines, ROS, and NO [10]. The cytotoxic effect of NO was evidenced by the decrease of cell viability in the LPS group as compared with vehicle control (Figure 1(c)). Cell viability after 20 h of SI cotreatment was greater than 95% of LPS group, implying the diminished NO production by SI (0.25 mg/mL) was not due to cell death. In comparison, ME (0.05–0.25 mg/mL) significantly attenuated LPS-induced cytotoxicity in a dose-dependent manner, indicating its cytoprotective effect may be attributable to its anti-inflammatory activity. On the other hand, BU enhanced LPS-mediated cell death so that the decrease in NO production by BU is likely due to its cytotoxic effect. In conclusion, the above results suggest that the anti-inflammatory principles of SI are most likely to be phenolic compounds and exist in the methanol fraction (ME).

### 3.3. Methanol Fraction (ME) Represses LPS-Induced iNOS Expression

**Figure 2:**
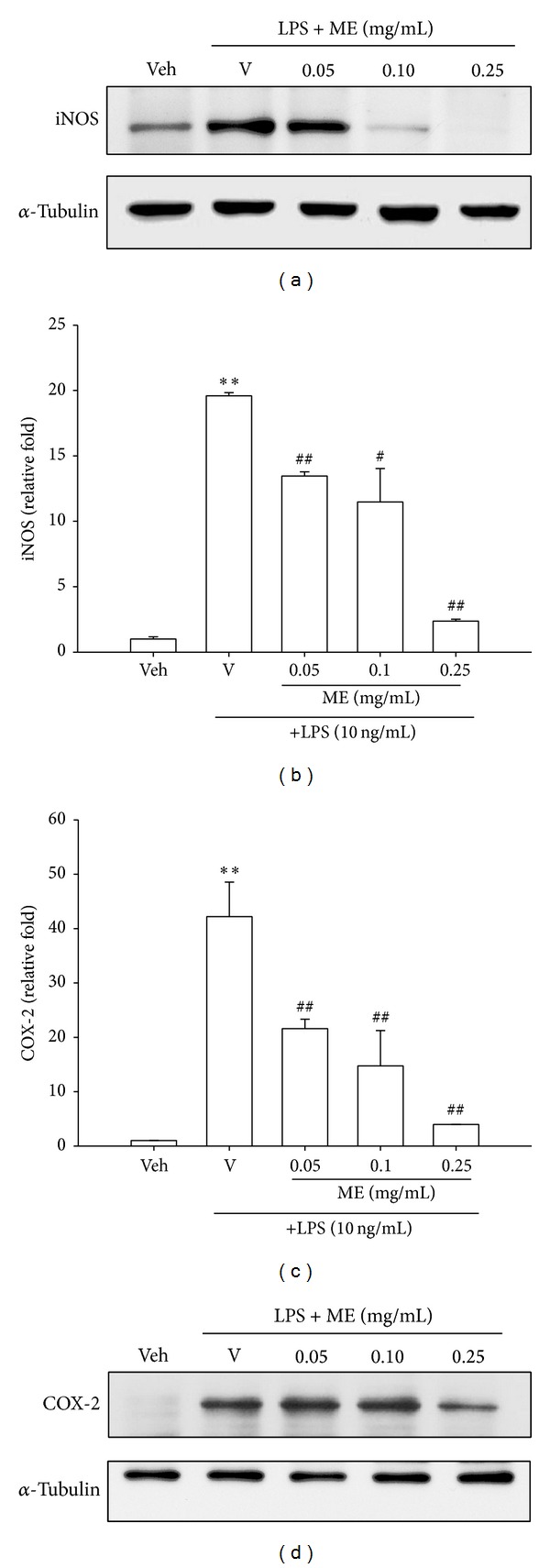
The methanol fraction of* Solanum integrifolium* (ME) inhibits LPS-induced iNOS and COX-2 expression in RAW264.7 macrophages. (a and d) RAW264.7 macrophages were cultured with vehicle (0.1% DMSO) or LPS (10 ng/mL) in the presence of the indicated concentrations of ME in 6-well plates for 16 h. Total cell lysates were prepared and the iNOS and COX-2 protein expressions were detected by Western blotting, as described in Section 2. The levels of *α*-tubulin in the total lysates serve as loading control. The blot is representative of at least three experiments. (b and c) RAW264.7 cells were cultured as described above for 12 h. Total RNA was prepared and the mRNA levels of iNOS and COX-2 were quantified by RT-Q-PCR, as described in Section 2. Data represent the mean ± SD of three independent experiments. **P < 0.01 represents significant differences compared with the vehicle control (without LPS). ^#^ *P* < 0.05; ^##^ *P* < 0.01 represent significant differences compared with the LPS-treated vehicle.

To determine whether ME exerts NO inhibition on activated macrophages by blocking inducible nitric oxide synthase (iNOS) expression, Western blot analysis was carried out on the whole cell lysates of RAW264.7 cells. It is found that RAW264.7 macrophages expressed a low level of iNOS protein and LPS (10 ng/mL) caused a dramatic increase after 16-hour treatment. ME (0.05–0.25 mg/mL) reduced the level of iNOS protein in a dose-dependent manner, and 0.1 mg/mL of ME inhibited iNOS protein to the level lower than that of vehicle control. *α*-Tubulin serves as a loading control to make sure equal protein was analyzed (Figure 2(a)).

The effects of ME on iNOS mRNA levels were further investigated. RT-Q-PCR analysis of the extracted mRNA revealed that treatment of RAW264.7 cells with LPS (10 ng/mL) for 12 h caused a 19.5-fold increase in iNOS mRNA expression, as compared with the vehicle control group (Figure 2(b)). ME (0.05–0.25 mg/mL), in conjunction with the stimuli, blocked this induction significantly and dose dependently. There was a slightly elevated iNOS mRNA (about 2-fold compared with vehicle control) for the group of 0.25 mg/mL ME, indicating ME regulated iNOS expression at both mRNA and protein levels.

### 3.4. Methanol Fraction (ME) Inhibits COX-2 Expression

Inducible cyclooxygenase (COX-2) expression increases significantly during an inflammatory response, resulting in high levels of prostaglandin E_2_ (PGE_2_) [4]. A variety of phenolic substances and crude plant extracts have been reported to inhibit the COX-2 expression [7, 11]. To examine whether ME also has such an action, COX-2 mRNA and protein expression were analyzed. It was found that incubation of cells with LPS (10 ng/mL) for 12 h increased COX-2 mRNA expression by about 43-fold, as compared with the vehicle. Cotreatment with ME (0.05–0.25 mg/mL) exerted a dose-dependent inhibition against LPS-stimulated COX-2 mRNA expression (Figure 2(c)). On the other hand, only 0.25 mg/mL ME significantly inhibited LPS-stimulated COX-2 protein expression, but lower concentrations (0.05 and 0.1 mg/mL) did not (Figure 2(d)). Compared with those for iNOS expression, this indicates that ME downregulates iNOS protein expression more effectively than COX-2 expression.

### 3.5. Methanol Fraction (ME) Inhibits LPS-Stimulated mRNA Expression of Proinflammatory Cytokines and Chemokines

**Figure 3:**
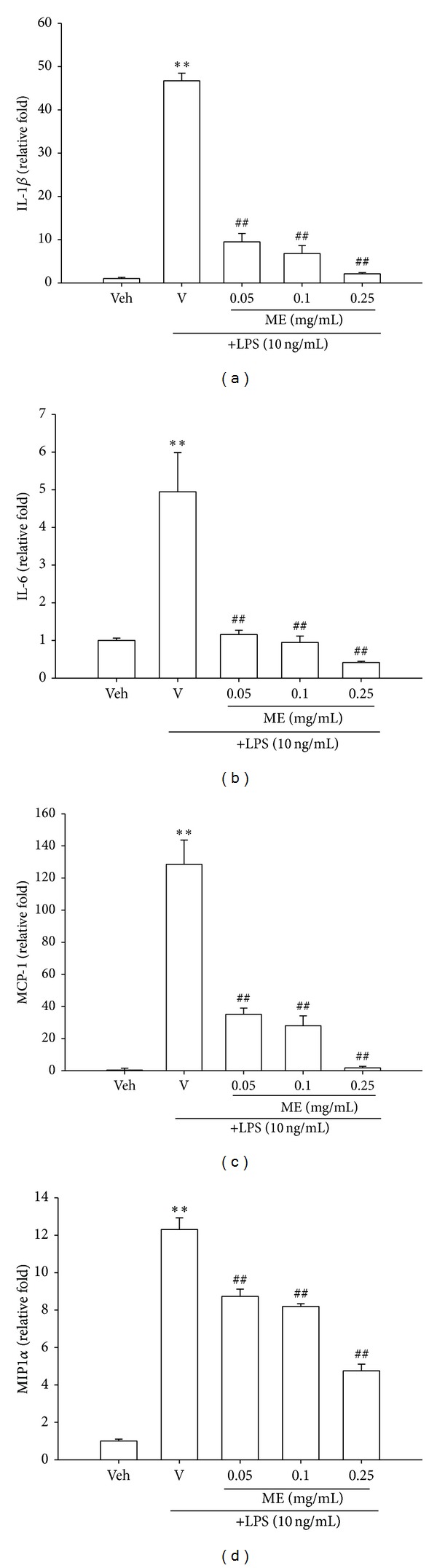
The methanol fraction of* Solanum integrifolium* (ME) inhibits LPS-induced mRNA expression of proinflammatory cytokines and chemokines in RAW264.7 macrophages. RAW264.7 macrophages were cultured with vehicle (0.1% DMSO) or LPS (10 ng/mL) in the presence of the indicated concentrations of methanol fraction (ME) in 6-well plates for 12 h. Total RNA was prepared and the mRNA levels of IL-1*β*, IL-6, CCL2/MCP-1, and CCL3/MIP1*α* were detected by RT-Q-PCR, as described in Section 2. Data represent the mean ± SD of three independent experiments. **P < 0.01 represents significant differences compared with the vehicle control (without LPS). ^##^ *P* < 0.01 represents significant differences compared with the LPS-treated vehicle.

The level and persistence of proinflammatory cytokines and chemokines play important roles in determining the extent of inflammation. It has been shown that transcriptomic changes are also reflected at the proteomic level in RAW264.7 cells [12, 13]. To investigate whether ME inhibits cytokine gene expression, we analyzed the changes of mRNA levels of IL-1*β*, IL-6, CCL2/MCP-1, and CCL3/MIP1*α* by RT-Q-PCR. IL-1*β* is an important primary inflammatory mediator produced in macrophages. Its mRNA expression can be induced slowly by LPS and has a long half-life [14]. Current study shows that IL-1*β* mRNA expression was elevated by 46.7-fold after 12 h of LPS (10 ng/mL) treatment and 0.05, 0.1, and 0.25 mg/mL ME inhibited IL-1*β* mRNA expression by 80%, 85%, and 95%, respectively (Figure 3(a)).

The cytokine interleukin-6 (IL-6) is one of the major proinflammatory cytokines produced by monocytes and macrophages. This cytokine is involved in autoimmune disorders and chronic inflammation and mediates the innate-adaptive immunity interface [15]. IL-6 mRNA expression was raised by 4.9-fold after 12 h of LPS (10 ng/mL) treatment, while treatment with ME as low as 0.05 mg/mL could completely block its upregulation (Figure 3(b)).

One of the most important chemokines known to regulate the migration and infiltration of monocytes/macrophages is CCL2 (CC chemokine ligand 2)/MCP-1 (monocyte chemoattractant protein-1). In macrophages, expression of the MCP-1 gene is stimulated by LPS [16] via the nuclear factor (NF)-*κ*B-dependent mechanism through activation of the LPS/TLR4/MyD88 signaling pathway [17]. A tremendous increase in CCL2/MCP-1 mRNA expression (by about 128-fold) was observed after 12-hour treatment with LPS (10 ng/mL). Cotreatment with ME effectively and dose dependently inhibited its induction, and complete inhibition was observed at 0.25 mg/mL (Figure 3(c)).

CCL3 (CC chemokine ligand 3)/MIP1*α* (macrophage inflammatory protein-1*α*) is secreted by activated macrophages and other inflammatory cells and has a range of diverse functions, such as chemotaxis, phagocytosis, and mediator release [18, 19]. High levels of CCL3/MIP1*α* promote inflammation and have been proposed to be involved in a broad range of diseases, from asthma to multiple sclerosis [20, 21]. CCL3/MIP1*α* mRNA was increased by 12.3-fold by LPS (10 ng/mL) after 12-hour treatment, and ME dose dependently attenuated its upregulation (Figure 3(d)). These results indicate that ME was effective in inhibiting mRNA expression of proinflammatory cytokines and chemokines.

### 3.6. Methanol Fraction (ME) Induces HO-1 Expression

**Figure 4:**
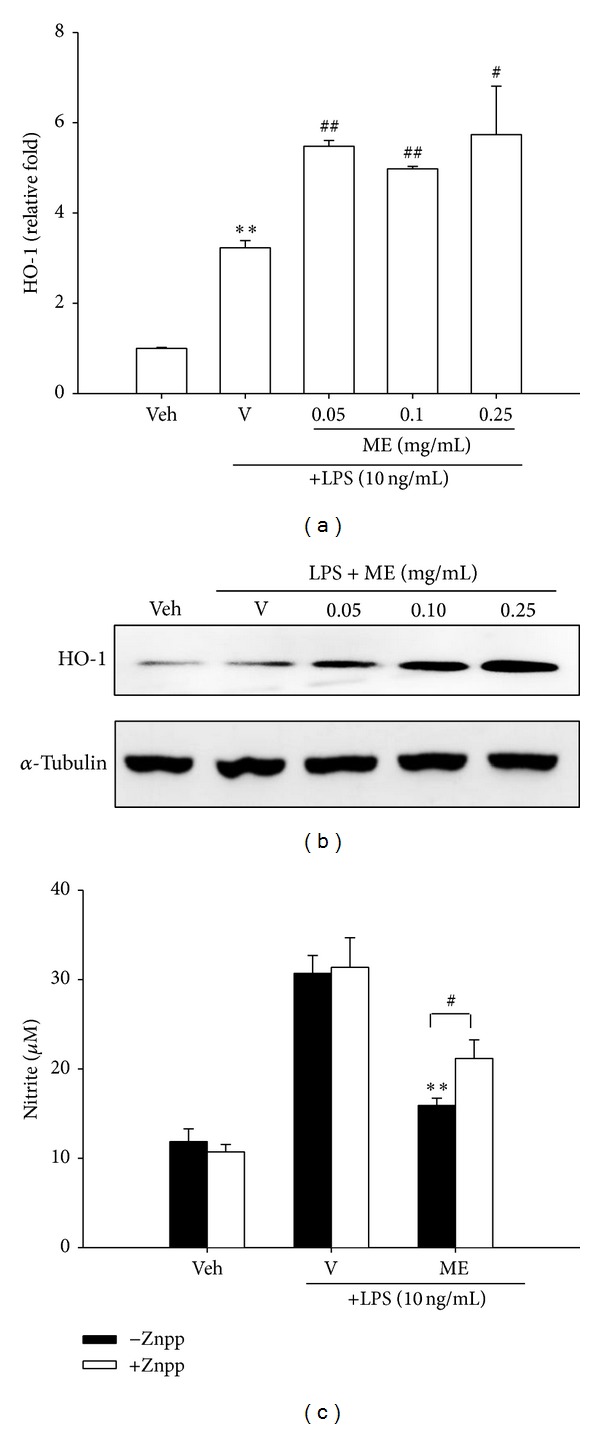
Involvement of HO-1 upregulation in the anti-inflammatory effect of the methanol fraction of* Solanum integrifolium* (ME). (a) RAW264.7 macrophages were cultured with vehicle (0.1% DMSO) or LPS (10 ng/mL) in the presence of the indicated concentrations of methanol fraction (ME) in 6-well plates for 12 h. Total RNA was prepared and HO-1 mRNA level was detected by RT-Q-PCR, as described in Section 2. Data represent the mean ± SD of three independent experiments. **P < 0.01 represents significant differences compared with the vehicle control (without LPS). ^#^ *P* < 0.05; ^##^ *P* < 0.01 represent significant differences compared with the LPS-treated vehicle. (b) RAW264.7 cells were cultured as described above for 16 h. Total cell lysates were prepared and HO-1 protein expression was detected by Western blotting, as described in Section 2. These blots are representative ones from one of three independent experiments. (c) RAW264.7 cells were treated with ME (0.1 mg/mL) and LPS (10 ng/mL) in the presence or absence of ZnPP (10 *μ*M) for 20 h, and the amount of NO produced in the medium was determined by the Griess reaction. Data were obtained from four independent experiments and are expressed as the mean ± SD. **P < 0.01 indicates significant differences from the respective LPS-treated group; ^#^ *P* < 0.05 indicates significant differences from Znpp untreated group.

Heme oxygenase 1 (HO-1) is a known NF-E2-related factor 2 (Nrf2) target gene, with established antioxidant and anti-inflammatory properties [22]. A growing body of evidence indicates that HO-1 induction counteracts inflammatory responses in macrophages [9, 23].  Figure 4(a) shows that treatment of RAW264.7 cells with LPS (10 ng/mL) for 12 h induced a moderate increase of HO-1 mRNA expression and this response was additive to ME. In parallel, LPS only slightly induced HO-1 protein expression, but addition of ME caused a synergic increase in HO-1 protein expression in LPS-stimulated macrophages (Figure 4(b)).

To further examine the anti-inflammatory role of HO-1, RAW264.7 cells were treated with ZnPP (zinc protoporphyrin IX), a potent competitive inhibitor of HO enzyme activity, for 30 min followed by ME (0.1 mg/mL) and then LPS (10 ng/mL) challenge for 20 h.  Figure 4(c) shows that the addition of ZnPP (10 *μ*M) did not affect NO release in vehicle control or LPS-treated cells. However, cotreatment of cells with ZnPP and ME in the presence of LPS significantly attenuated ME's inhibitory action against LPS-induced NO production, indicating that HO-1 activity participated in the anti-inflammatory mechanism of ME.

### 3.7. Methanol Fraction (ME) Upregulates LPS-Activated Extracellular Signal-Regulated Kinase (ERK) Signaling Pathway

**Figure 5:**
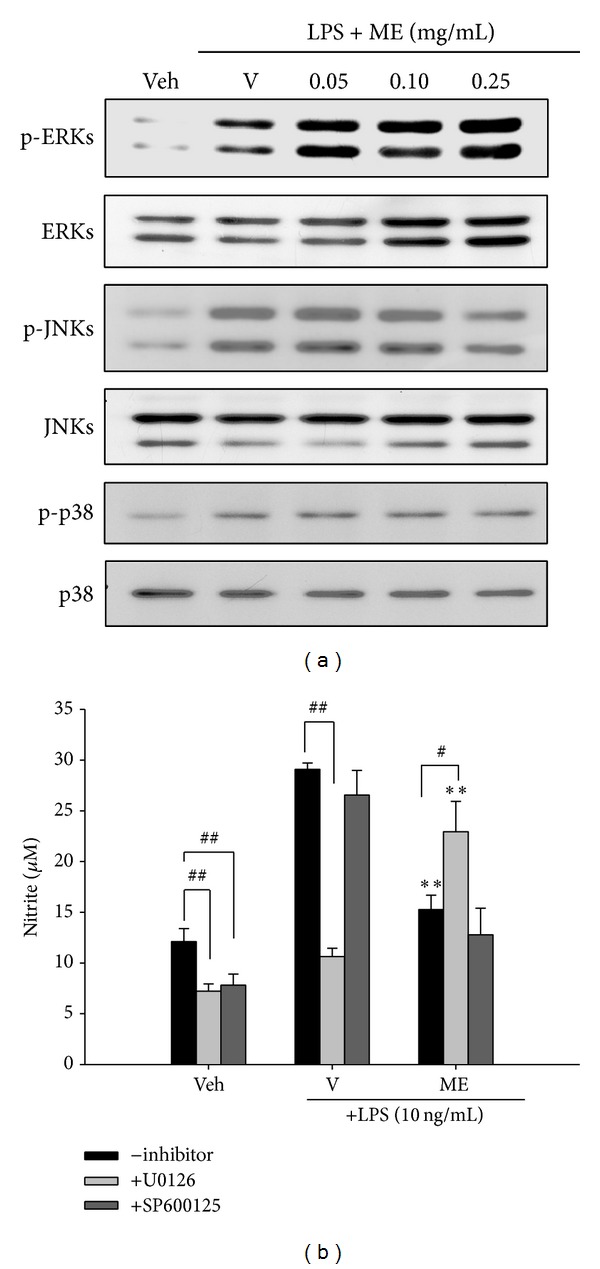
Contributions of MAPKs on the anti-inflammatory action of the methanol fraction of* Solanum integrifolium* (ME). (a) RAW264.7 macrophages were cultured with vehicle (0.1% DMSO) or LPS (10 ng/mL) in the presence of the indicated concentrations of methanol fraction (ME) for 1 h. Total cell lysates were prepared and the phospho-ERK, JNK, and P38 MAPK were detected by Western blotting using antibodies specific for phosphoprotein. The same blots were then stripped and reprobed with antibodies specific for pan protein. These blots are representative ones from one of three independent experiments. (b) RAW264.7 cells were pretreated for 30 min with inhibitor (U0126 and SP600125, 10 *μ*M) followed by ME (0.1 mg/mL) and then LPS (10 ng/mL) challenge for 20 h, and the amount of NO produced in the medium was determined by the Griess reaction. Data were obtained from four independent experiments and are expressed as the mean ± SD. **P < 0.01 indicating significant differences from the respective LPS-treated group; ^##^ *P* < 0.01; ^#^ *P* < 0.05 indicate significant differences from respective inhibitor-untreated group.

Mitogen-activated protein kinases compose a family of protein kinases that play an essential role in relaying extracellular signals from the cell membrane to the nucleus via a cascade of phosphorylation events [24]. The impact of ME on LPS-stimulated activation of extracellular signal-regulated kinase (ERK), c-Jun NH_2_-terminal kinase (JNK), and p38 MAPK was thus studied. RAW264.7 cells were cultured in the presence of LPS (10 ng/mL) and ME for 1 h, and cell lysates were immunoblotted with pan-/phosphospecific antibodies as described in Section 2.  Figure 5(a) shows that phospho-ERK1/2 (44/42 kDa) and phospho-JNKs (46/54 kDa) were strongly induced by LPS, while phospho-p38 MAPK (43 kDa) was only modestly increased. The same blots were stripped and reprobed with antibodies that detected total levels of ERK1/2, JNKs, and p38 MAPK, demonstrating an equal amount of loading with regard to the total amount of proteins. LPS-induced ERK activation was enhanced by ME synergistically, but JNK activation was only slightly attenuated by high concentration of ME, and no change was observed in p38 MAPK activation.

To determine whether modulation of ERK and JNK activation relates to ME-mediated anti-inflammatory activity, RAW264.7 cells were pretreated with inhibitor of each pathway, U0126 and SP600125, for 30 min and then incubated with 0.1 mg/mL ME and LPS (10 ng/mL) for 20 h before analyzing NO production.  Figure 5(b) shows that the addition of U0126 (10 *μ*M), a specific inhibitor of mitogen-activated protein kinase kinase (MEK), that is an upstream activator of ERK1/2, inhibited endogenous and LPS-mediated NO production, confirming the involvement of ERK1/2 as an important signaling element in NO synthesis in mouse macrophages [25]. Furthermore, the inhibitory effect of ME on NO production was significantly attenuated by U0126, as compared with no inhibitor control (*P* < 0.05). This result indicates that upregulation of ERK signaling pathway participates in ME's anti-inflammatory action. In contrast, JNK inhibitor SP600125 (10 *μ*M) inhibited endogenous NO production but had no effect on LPS-induced NO production in the presence or absence of ME. This suggests JNK signaling may not be involved in the anti-inflammatory action of ME.

## 4. Discussion

Phenolic compounds are considered to possess anti-inflammatory properties and therefore were proposed as an alternative natural approach to prevent or treat chronic inflammatory diseases. The present study shows for the first time that the highest total phenolic content corresponds to the most potent anti-inflammatory activity for the extract of* S. integrifolium* green fruit, an indigenous medicinal vegetable consumed by Taiwanese aborigines. Among the partition fractions of the crude ethanol extract, the methanol fraction (ME) has the highest phenolic content, the strongest inhibitory effect against nitric oxide (NO) release, and the most potent cytoprotective activity in LPS-treated RAW264.7 cells. Data from the Western blot analysis carried out in this work further demonstrates that ME suppresses iNOS expression in LPS-stimulated macrophages. This result indicates that ME inhibits NO generation in LPS-activated cells principally through repression of iNOS expression. The results of RT-Q-PCR further suggests that reduction in iNOS proteins may primarily result from a decrease in iNOS mRNA.

Prostaglandins (PGs) play a key role in the generation of the inflammatory response. Cyclooxygenase (COX) catalyzes the first two steps in the biosynthesis of PGs, such as prostacyclin and thromboxane, from arachidonic acid. There are two isoforms of COXs, constitutively expressed COX-1 and inflammation-induced COX-2. Specific inhibition of COX-2 expression has been of great interest in developing anti-inflammatory drugs [4]. This study shows that ME inhibits LPS-mediated COX-2 mRNA and protein expression dose dependently, although its effects on them are weaker than those seen for iNOS.

The stimulation of Toll-like receptor 4 (TLR4) by lipopolysaccharide (LPS) induces the release of critical proinflammatory cytokines that are necessary to activate potent immune responses [26]. Chemokines are critically involved in leukocyte migration but also affect the biology of leukocytes in several ways. CCL2/MCP-1 is the prototype of the CC chemokine subfamily and exhibits the most potent chemotactic activity for monocytes [2]. CCL3/MIP1*α* not only mediates macrophage chemotaxis, but also significantly enhances differentiation of primed CD8(+) T cells into effector cells and their release into circulation, thus potentiating effective migration to the site of infection [27]. It has been shown that the patterns of mRNA and proteins of cytokine/chemokine are similar in activated RAW264.7 cells [12, 28]. This study shows that LPS stimulates significant increases in transcription of IL-1*β*, IL-6, CCL2/MCP-1, and CCL3/MIP1*α*. ME treatment effectively represses LPS-stimulated mRNA expression, indicating its strong anti-inflammatory efficacy.

The nuclear factor erythroid 2-related factor 2 (Nrf2) is an emerging regulator of cellular resistance to oxidants [29, 30]. Nrf2 directly affects the homeostasis of ROS and RNS by regulating the antioxidant defense systems through inducing the expression of Phase II enzyme genes, such as HO-1, GCL, and GPx. Various reports support the view that HO-1 induction results in the downregulation of inflammatory responses [9, 23, 31, 32]. Current data reveal that ME stimulates mRNA and protein expression of HO-1. HO-1 activity is further proved to be involved in ME's ant-inflammatory action because the addition of Znpp, the competitive inhibitor, substantially attenuates ME's inhibitory activity against LPS-stimulated NO release.

The key downstream pathway for LPS-induced signaling events is the MAPK cascade, which leads to several functional responses. The MAPK family is composed of the ERK, JNK, and p38 MAPK pathways. The activated MAPKs are responsible for phosphorylating and activating numerous transcription factors, which function to stimulate the synthesis of various proinflammatory cytokines, iNOS and COX-2. Current study shows that LPS treatment leads to activation of all three MAPK pathways, and cotreatment with ME further activates ERK but slightly inhibits JNK activation. The use of specific inhibitors for MAPK pathways confirms the involvement of ERK pathway, but not of JNK pathway, in the anti-inflammatory action of ME. These data are in good agreement with other reports that numerous compounds, both natural and man-made ones, inhibit proinflammatory mediators through upregulation of HO-1 and ERK signaling [33–35].

There is no literature regarding the chemical composition of scarlet eggplant could be found, while phytochemical investigation on the eggplant (*Solanum melongena* L.) indicated that N-caffeoylputrescine, 5-caffeoylquinic acid, and 3-acetyl-5-caffeoylquinic acid made up the bulk of its total phenolics [36]. Isolation of the active constituents of ME by silica gel column chromatography is currently underway. Preliminary ^1^H NMR (500 MHz) and ^13^C NMR (125 MHz) spectral data indicate ME also contain caffeoylquinic acid derivatives.

## 5. Conclusions

In conclusion, this research shows for the first time the correlation between phenolic content and anti-inflammatory activity of green fruit of* S. integrifolium*. ME, the highest phenolic fraction, reduces NO production, iNOS protein, and mRNA expression in LPS-activated RAW264.7 cells. It also represses the expression of LPS-induced COX-2, proinflammatory cytokines, IL-1*β* and IL-6, and chemokines, CCL2/MCP-1 and CCL3/MIP1*α*. The upregulation of HO-1 expression and activation of ERK phosphorylation contribute, at least in part, to the anti-inflammatory action of ME.

## Figures and Tables

**Table 1:** Primary antibodies used in Western blotting.

Antibody	Company	Catalog number
*α*-Tubulin	Sigma	T 6199
NOS	Cell Signaling	2977
COX-2	Santa Cruz	Sc-166475
HO-1	Stressgen	SPA-895
ERK	Cell Signaling	4695
p-ERK	Cell Signaling	4370
p38	Cell Signaling	9212
p-p38	Cell Signaling	9215
JNK	Cell Signaling	9258
p-JNK	Cell Signaling	4668

**Table 2:** Primer pairs used in RT-Q-PCR.

Gene	Primers	Amplicon (bp)
*β*-Actin	GGCTGTATTCCCCTCCATCG CCAGTTGGTAACAATGCCATGT	154
iNOS	GTTCTCAGCCCAACAATACAAGA GTGGACGGGTCGATGTCAC	127
COX-2	TGAGCAACTATTCCAAACCAGC GCACGTAGTCTTCGATCACTATC	74
IL-1*β*	TTCAGGCAGGCAGTATCACTC GAAGGTCCACGGGAAAGACAC	75
IL-6	TAGTCCTTCCTACCCCAATTTCC CGCACTAGGTTTGCCGAGTA	153
CCL2/MCP-1	TTAAAAACCTGGATCGGAACCAA GCATTAGCTTCAGATTTACGGGT	121
CCL3/MIP1*α*	TTCTCTGTACCATGACACTCTGC CGTGGAATCTTCCGGCTGTAG	100
HO-1	AAGCCGAGAATGCTGAGTTCA GCCGTGTAGATATGGTACAAGGA	100
